# NOTUM is a potential pharmacodynamic biomarker of Wnt pathway inhibition

**DOI:** 10.18632/oncotarget.7157

**Published:** 2016-02-03

**Authors:** Babita Madan, Zhiyuan Ke, Zheng Deng Lei, Frois Ashley Oliver, Masanobu Oshima, May Ann Lee, Steve Rozen, David M. Virshup

**Affiliations:** ^1^ Program in Cancer and Stem Cell Biology, Duke-NUS Medical School, Singapore; ^2^ Experimental Therapeutics Centre, A*STAR, Biopolis, Singapore; ^3^ Division of Genetics, Cancer Research Institute, Kanazawa University, Kanazawa, Japan; ^4^ Department of Pediatrics, Duke University, Durham, NC, USA

**Keywords:** Wnt signaling, Notum, Biomarker, PORCN inhibitor

## Abstract

Activation of Wnt signaling due to Wnt overexpression or mutations of Wnt pathway components is associated with various cancers. Blocking Wnt secretion by inhibiting PORCN enzymatic activity has shown efficacy in a subset of cancers with elevated Wnt signaling. Predicting response to upstream Wnt inhibitors and monitoring response to therapeutics is challenging due to the paucity of well-defined biomarkers. In this study we identify Notum as a potential biomarker for Wnt driven cancers and show that coordinate regulation of *NOTUM* and *AXIN2* expression may be a useful predictor of response to PORCN inhibitors. Most importantly, as NOTUM is a secreted protein and its levels in blood correlate with tumor growth, it has potential as a pharmacodynamic biomarker for PORCN and other Wnt pathway inhibitors.

## INTRODUCTION

Wnts are secreted morphogens essential for embryonic development and tissue homeostasis [1, 2]. Dysregulated Wnt signaling causes various developmental abnormalities and diseases such as cancers, fibrosis and osteoporosis [3]. Interaction of Wnts with the cell surface receptors Frizzled, low-density lipoprotein receptor-related proteins 5 and 6 (LRP 5/6) and G protein coupled receptors activates both β-catenin dependent and β-catenin independent pathways. The mono-unsaturated palmitoleic acid conjugated to a serine residue conserved in all the Wnts is essential for the engagement of Wnts with its receptors Frizzleds as the palmitoleate group inserts into a hydrophobic groove of the Fzd cysteine rich domain [4]. This palmitoleation of Wnts mediated by an ER resident enzyme Porcupine (PORCN) is also essential for their secretion. Mutating the conserved serine residue in Wnt, or inhibition of PORCN enzymatic activity prevents the interaction of Wnts with their carrier protein Wntless (WLS) and hence blocks Wnt secretion [4–6].

Wnt/β-catenin signaling regulates expression of its own regulators, including the negative regulators of Wnt/β-catenin signaling, *AXIN2* and *NOTUM*. *NOTUM* encodes a secreted Wnt antagonist that regulates WNT/Wg activity gradients in Drosophila imaginal discs and in vertebrate embryogenesis [7–9]. While originally proposed to cleave glycosylphosphatidylinositol linkages, more recently NOTUM has been shown to be a carboxyl oxoesterase that functions as a Wnt antagonist by deacylating Wnts [8, 10]. The crystal structure demonstrates that NOTUM has a large hydrophobic pocket that can accommodate *cis*-unsaturated fatty acid palmitoleate [10]. The glycosaminoglycan binding sites on NOTUM facilitate its interaction with heparin sulphate glycoproteins, glypicans that act as scaffolds to co-localize NOTUM with its substrate Wnt proteins.

Preventing the secretion of Wnts by inhibiting the PORCN enzymatic activity inhibits both canonical and non-canonical Wnt signaling; hence PORCN inhibitors have shown high efficacy in the subset of cancers driven by high Wnt signaling including molecularly defined colorectal cancers with R-spondin translocations and pancreatic cancers with RNF43 mutations [11]. As these inhibitors advance to the clinical trials, identification of predictive and pharmacodynamic markers is imperative for the selection and treatment of patients. In this study we show that regulation of *NOTUM* expression correlates with sensitivity to PORCN inhibitors. We also show that NOTUM can be a potential pharmacodynamic biomarker for Wnt pathway inhibitors.

## RESULTS AND DISCUSSION

### NOTUM is a pharmacodynamic biomarker for PORCN inhibitors *in vitro*

**Figure 1:**
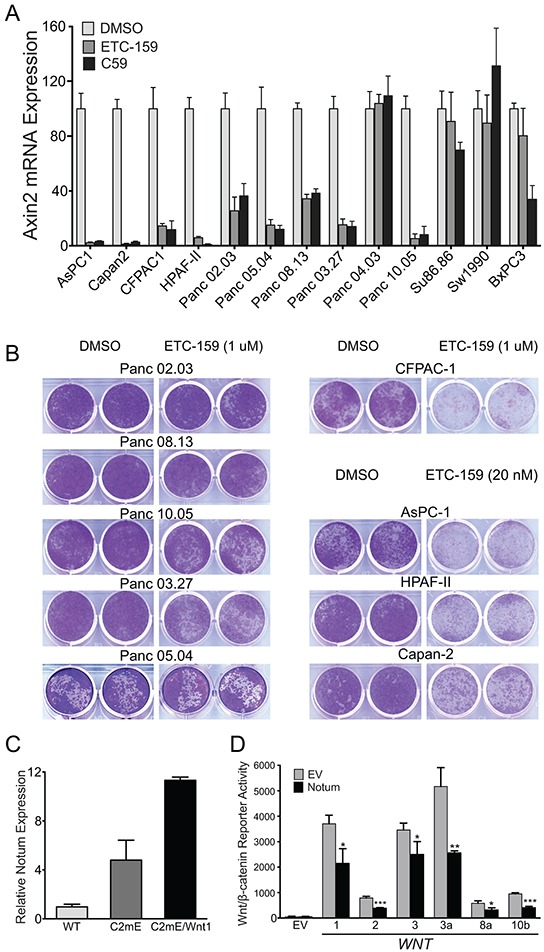
AXIN2 gene expression is a poor predictor of PORCN inhibitor sensitivity **A. *AXIN2 expression is widely suppressed in pancreatic cell lines treated with Wnt-C59 and ETC-159:*** Pancreatic cancer cell lines were treated with 100 nM ETC-159 or 100 nM Wnt-C59 for 24h. Total RNA was isolated and *AXIN2* expression was analyzed by qRT-PCR. Expression of *AXIN2* normalized to 18SrRNA in the ETC-159 or Wnt-C59 treated cells is represented as the percentage of respective DMSO treated controls. **B. *ETC-159 blocks foci formation in a subset of pancreatic cell lines*:** 1000-3000 cells of the indicated pancreatic cell lines were plated in 24 well plates and treated with ETC-159 (10 nM to 1 μM). Cell number and the duration for treatment were determined based on the growth rate of each line. Following methanol fixation the cells were stained with crystal violet. **C. *Expression of Notum is elevated in Gan mice*:** Total RNA was isolated from gastric tissue of K19-C2mE (C2mE) and K19-C2mE/Wnt1 (Gan) mice and *Notum* expression was measured by qRT-PCR. Expression was normalized to *Hprt*, n=5 mice /group. **D. *NOTUM inhibits Wnt/β-catenin reporter activity induced by diverse Wnts:*** HT1080 cells were transiently transfected with the Super8xTOPFLASH (STF), NOTUM and the indicated Wnt expression plasmids. The reporter activity was normalized to mCherry expression, a control for transfection efficiency. Bars represent the mean ± SD. *** p ≤0.001, ** p ≤0.01, * p ≤0.05.

*AXIN2* expression has been used extensively as readout for Wnt pathway activity. To identify cancers sensitive to Wnt secretion inhibitors, 13 pancreatic cell lines were treated with 2 structurally unrelated porcupine inhibitors Wnt-C59 (IC_50_ = 0.1 nM) and ETC-159 (IC_50_ = 3 nM) [6, 11] for 24 h. In 9 cell lines, the PORCN inhibitors reduced *AXIN2* mRNA expression by more than 50%, suggesting the presence of endogenous autocrine Wnt signaling (Figure 1A). We next tested if this Wnt autocrine signaling was important for growth of these 9 cell lines in which PORCN inhibition reduced *AXIN2* expression. We tested the effect of ETC-159 on their proliferation after low-density plating. However, drug-induced downregulation of *AXIN2* expression did not correlate well with drug-induced inhibition of proliferation. The proliferation of 4 cell lines was inhibited, while 5 cell lines were not affected even in high concentration (1 μM) of ETC-159 (Figure 1B).

As the drug-dependent repression of *AXIN2* gene expression correlated poorly with inhibition of proliferation after low density plating, we investigated additional Wnt/β-catenin target genes that could serve as predictive or pharmacodynamic biomarkers. Analysis of the Singapore and Australian gastric cancer tissue databases for expression of β-catenin target genes revealed that *NOTUM* mRNA expression highly correlated (correlation ≥ 0.64) with *AXIN2* mRNA expression (Table 1A–1B). Consistent with this, Wnt-driven gastric tumors from *K19-Wnt1/C2mE* mice [14] that overexpress *Wnt1*, *Ptgs2* and *Ptges* in the gastric epithelium, also had high *Notum* mRNA expression (Figure 1C). *NOTUM* is a Wnt/β-catenin target gene [15] reported to negatively regulate Wnt signaling in zebrafish [7] and is potentially valuable as a biomarker because it is a secreted protein. We confirmed that ectopic expression of human NOTUM inhibits signaling activity driven by diverse Wnts (Figure 1D).

**Table 1:** *AXIN2* correlated genes

(A) Singapore Gastric cancer dataset				
Probeset	Correlation	P value	FDR	Gene
**224176_s_at**	**0.92**	**0**	**0**	**AXIN2**
**222696_at**	**0.86**	**0**	**0**	**AXIN2**
**222695_s_at**	**0.74**	**0**	**0**	**AXIN2**
**228649_at**	**0.64**	**0**	**0**	**NOTUM**
229481_at	0.63	0	0	LOC283859
**224498_x_at**	**0.62**	**0**	**0**	**AXIN2**
235845_at	0.59	0	0	SP5
207607_at	0.59	0	0	ASCL2
209494_s_at	0.56	0	0	PATZ1
226360_at	0.55	0	0	ZNRF3
229215_at	0.54	2.22E-16	1.01E-12	ASCL2
218704_at	0.53	4.44E-16	1.87E-12	RNF43
208608_s_at	0.52	2.89E-15	1.13E-11	SNTB1
240211_at	0.51	6.22E-15	2.26E-11	LOC100130468

**(B) Australian Gastric cancer dataset**				
Probeset	Correlation	P value	FDR	Gene
224176_s_at	0.93	0	0	**AXIN2**
222696_at	0.88	0	0	**AXIN2**
229481_at	0.69	3.36E-11	6.12E-07	LOC283859
222695_s_at	0.68	6.73E-11	9.19E-07	**AXIN2**
205983_at	0.66	4.18E-10	4.57E-06	DPEP1
1555497_a_at	0.66	6.65E-10	6.05E-06	CYP4B1
208121_s_at	0.65	1.14E-09	8.89E-06	PTPRO
**228649_at**	**0.65**	**1.42E-09**	**9.69E-06**	**NOTUM**
210550_s_at	0.64	1.88E-09	1.14E-05	RASGRF1
231814_at	0.62	9.04E-09	4.75E-05	MUC12
218704_at	0.62	1.14E-08	5.17E-05	RNF43
205555_s_at	0.61	1.43E-08	6.03E-05	MSX2

**Figure 2:**
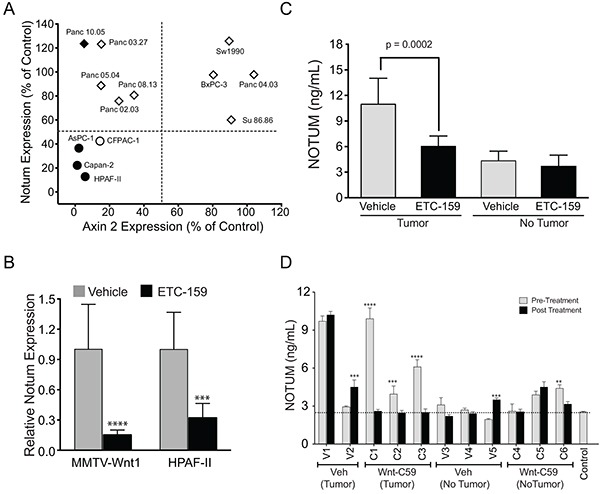
**A. Combined inhibition of *NOTUM* and *AXIN2* expression predicts sensitivity to PORCN inhibitors:** *AXIN2* and *NOTUM* expression was assessed in cell lines exposed to 100 nM ETC-159 for 24 hours. RNF43 mutant (filled symbols) and wild-type (open symbols) cell lines. ETC-159 sensitive (circle) or insensitive (diamond) cell lines. **B. ETC-159 treatment decreases *Notum* expression in MMTV-Wnt1 allografts and HPAF-II tumors:** RNA isolated from vehicle or ETC-159 treated MMTV-Wnt1 tumors (n=9 in each group) or HPAF-II tumors (n=6 in each group) was assessed by RT-qPCR. Data presented are mean ± SD, Data were analyzed using unpaired t-test, **** p ≤0. 0001, *** p ≤0. 001. **C. NOTUM protein is decreased in plasma of ETC-159 treated mice:** Control BALB/c nude mice or mice with established MMTV-Wnt1 tumors were treated daily for 10 days with ETC-159 (30 mg/kg). Blood was collected 6 hours after the last dose and plasma NOTUM levels were measured using ELISA. The graph shows group means ± SD. n=10 for tumor bearing mice in each group and n=4 for control BALB/c nude mice. **D. Wnt-C59 treatment decreases NOTUM protein levels in MMTV-Wnt1 mice:** Plasma NOTUM levels were measured in control (C57/Bl6, n=4) or transgenic MMTV-Wnt1 mice before initiating the treatment and after 3 days of treatment with 10 mg/kg Wnt-C59 as measured by ELISA. Data represents plasma NOTUM levels of individual mice. Dotted line represents NOTUM levels in control mice. V=vehicle-treated mice, C=Wnt-C59-treated mice. *** p ≤0.001, **** p ≤0.0001.

To determine if *NOTUM* expression was indeed regulated by Wnt signaling, we measured the abundance of *NOTUM* mRNA in the same 13 cell lines following PORCN inhibition (Figure 2A). *NOTUM* transcript expression was suppressed by PORCN inhibition in a subset of the cells. Notably, only the cell lines in which both *NOTUM* and *AXIN2* were suppressed by more than 50% by PORCN inhibition were growth-inhibited by ETC-159 (Figure 1B). Downregulation of *NOTUM* expression strongly associates with response to *PORCN* inhibitor, p value = 0.0028 (Wilcoxon rank sum test). Of note, 3 of these 4 cell lines have loss of function mutations in RNF43, which sensitizes cells to Wnts [11, 16]. Notably not all cell lines with RNF43 mutation such as Panc10.05 (M18fs) were sensitive to PORCN inhibition despite decreased *AXIN2* expression. These data suggest that coordinated reduction of *AXIN2* and *NOTUM* mRNA expression may be a better predictor of Wnt addiction and response to PORCN inhibitors than each gene individually.

### Notum is a pharmacodynamic biomarker for PORCN inhibitors *in vivo*

We identified *NOTUM* gene expression as a good predictor for response to PORCN inhibitors in pancreatic cell lines *in vitro*. To test if its expression also correlated with the inhibition of Wnt signaling pathway in tumors *in vivo*, we evaluated *Notum* gene expression in Wnt dependent cancer models. Mice carrying an MMTV LTR-*Wnt1* transgene have marked overexpression of *Wnt1* in the mammary gland, driving hyperplasia and an eventual development of adenocarcinomas [17]. Tumor fragments from MMTV-Wnt1 tumors were orthotopically transplanted into the 4^th^ mammary fat pad of BALB/c nude mice. We have previously reported that once daily gavage dosing of ETC-159 significantly inhibited the growth of these tumors [11]. *NOTUM* was high in the tumors from the vehicle-treated mice and was decreased significantly in the ETC-159 treated mice (Figure 2B). We then tested a second Wnt-dependent human pancreatic cell line. We and others have shown that PORCN inhibition effectively prevents the growth of HPAF-II tumors, a pancreatic cancer cell line with an RNF43 mutation [11, 16]. A remarkable decrease in *NOTUM* expression was observed 56 hours after the start of ETC-159 treatment of established HPAF-II orthotopic pancreatic xenografts (Figure 2B). Taken together our data demonstrate that *NOTUM* expression is a useful predictor of response to PORCN inhibitors *in vivo*.

NOTUM is a secreted protein and therefore has the potential to be used as pharmacodynamic biomarker to monitor therapeutic response. We analyzed the protein levels of NOTUM in the plasma from the MMTV-*Wnt1* mice pre-and post-PORCN inhibitor treatment (Figure 2C & 2D). Basal levels of NOTUM in the BALB/c nude mice and the MMTV-*Wnt1* transgenic mice were low. NOTUM protein abundance in the plasma increased when the mice develop tumors spontaneously (MMTV-*Wnt1* transgenic mice, Figure 2D) or when tumors are established in BALB/c nude mice (Figure 2D). Following treatment with two independent PORCN inhibitors, ETC-159 (BALB/c nude mice, Figure 2C) or Wnt-C59 (MMTV-Wnt1 transgenic, Figure 2D) the levels of NOTUM in the blood decreased considerably to those seen in the control mice. These results demonstrate that NOTUM may be a useful pharmacodynamic marker for Wnt pathway inhibitors.

A subset of cancers are dependent on Wnt signaling, and can in part be identified by the presence of mutations that increase cellular sensitivity to Wnts [18]. New therapies that target Wnt signaling show activity in genetically identified Wnt-addicted cancers. One key feature of the response to these new therapies is differentiation, rather than apoptosis. This may account for the slow regression of these tumors in pre-clinical models [11, 16, 19]. It would be helpful to have more rapid readouts of tumor response. Measurement of changes in secreted proteins is one approach that is useful in other settings [20]. Here we demonstrate that PORCN inhibitors inhibit the Wnt target gene NOTUM. In addition, NOTUM protein can be detected in the blood of mice bearing Wnt-dependent MMTV-Wnt1 tumors and its abundance drops significantly upon therapy. These studies suggest that it would be useful to develop highly sensitive assays for circulating NOTUM protein and assess their utility in patients with Wnt-driven cancers.

## MATERIALS AND METHODS

### RNA isolation and qRT-PCR

All the cell lines were obtained from ATCC and were tested free of mycoplasma contamination. Total RNA was isolated from the cell lines or tumors using RNAeasy kit (Qiagen). RNA was reverse transcribed with iScript reverse transcriptase (BioRAD) and real time quantitative PCR (qPCR) was performed with SsoFast™ EvaGreen® assay Supermix from BioRad. HPRT was used as housekeeping gene. The primers used are *Axin2* F: 5′-CTCCCCACCTTGAATGAAGA-3′, R: 5′-TGGCTGGTGCAAAGACATAG-3′; *Hprt* F: 5′-CCT CACTGCTTTCCGGAGCGG-3′ R: 5′-ATCGCTAATCA CGACGCTGGGA-3′; *Notum* F: 5′-GGAAGGCCA GTG GCTATACATC-3′, 5′-GTCCGTCCAATAGCT CC GTATG-3′.

### TOPFLASH assays

HT1080 cells were transfected in 24-well plates with 50 ng Wnt, 100 ng mCherry, without or with Notum and 550 ng Super 8x TOPFLASH plasmids (a gift from Randall T Moon). 24 h after transfection, the cells were washed with PBS and lysed in 0.6% NP40 in PBS containing protease inhibitors. Super8xTOPFLASH reporter activity was measured using firefly luciferase substrate (Promega, Madison WI). The reporter activity was normalized to the transfection efficiency as determined using mCherry expression [12].

### Animal care

MMTV-Wnt1 mice were obtained from Jackson Laboratories (Bar Harbor, Maine) and were maintained by backcrossing to C57BL/6. BALB/c nude mice were purchased from InVivos, Singapore. The Duke-NUS Institutional Animal Care and Use Committee approved all the animal studies for compliance with regulations. Animals were housed in standard cages and were allowed access *ad libitum* to food and water.

### Tumor implantation, treatment of mice and analysis of plasma

Mouse xenograft models were established by subcutaneous implantation of MMTV-Wnt1-derived solid tissue fragments in BALB/c nude mice. Following development of palpable tumors, treatment was initiated. Mice were treated with ETC-159 formulated in 50% PEG400 (vol/vol) in water and administered by oral gavage at a dosing volume of 10 μL/g body weight. At sacrifice, blood was collected and plasma was isolated. Notum levels in the plasma were measured using ELISA kit from CusABio (CSB-EL015955HU) following manufacturer's protocol.

### Microarray data analysis

To identify correlation between *AXIN2* and other genes, expression data from 201 gastric tumors from Singapore (GSE15459 and GSE34942) and 70 gastric tumors from Australia (GSE35809) was analyzed [13]. *AXIN2* expression was calculated as an average expression value of its corresponding probesets (222695_s_at, 222696_at, 224176_s_at, and 224498_x_at). Pearson correlation coefficient was calculated for each probeset of the gene against the average expression level of *AXIN2*. p-values were adjusted to account for multiple testing using the Benjamini-Hochberg (FDR) correction.

### Data analysis

Data was analyzed using Prism v5.0 (GraphPad). Significance for all tests was set at p ≤ 0.05 unless otherwise stated.
